# Task-Dependent Neural Activity in the Posterior Parietal Cortex Is Associated with Better Balance in Adults with Acquired Brain Injury

**DOI:** 10.3390/brainsci15101049

**Published:** 2025-09-26

**Authors:** Jesus A. Hernandez-Sarabia, Arlene A. Schmid, Jaclyn A. Stephens

**Affiliations:** 1 Department of Neuroscience, Tarleton State University, Stephenville, TX 76401, USA; 2 Department of Health and Human Performance, Tarleton State University, Stephenville, TX 76401, USA; 3 Department of Occupational Therapy, Colorado State University, Fort Collins, CO 80523, USA; arlene.schmid@colostate.edu; 4 Columbine Health Systems Center for Healthy Aging, Colorado State University, Fort Collins, CO 80523, USA; 5 Translational Neurological Laboratory, Colorado State University Spur Campus, Denver, CO 80216, USA; 6 Department of Health and Exercise Science, Colorado State University, Fort Collins, CO 80523, USA; 7 Molecular Cellular, and Integrative Neuroscience Program, Colorado State University, Fort Collins, CO 80523, USA

**Keywords:** stroke, traumatic brain injury, postural control, brain imaging, functional near-infrared spectroscopy

## Abstract

**Background/Objectives**: There is scarce evidence on the neural underpinnings of balance in people with chronic acquired brain injury (ABI). Thus, the objective was to measure this in adults with ABI during four balance tasks and to examine the relationship between neural activity and balance performance. **Methods**: Twenty-seven adults with chronic ABI (Age (M ± SD): 51.30 ± 18.67 years, 18 females) were included in this study. Functional near-infrared spectroscopy (fNIRS) was used to measure task-dependent neural activity, which was quantified using oxygenated hemoglobin (HbO) beta values. A force plate was used to measure balance performance, quantified as the amount of sway. One-sample t-tests were used to test for significant task-dependent neural activity during each balance task (HbO > 0) at each fNIRS channel. Pearson’s correlations were used to test for relationships between fNIRS channels with significant task-dependent activity and balance performance. **Results**: Significant task-dependent neural activity was observed in an fNIRS channel situated over the right superior parietal lobe, *p* = 0.039, along with 4 channels over the right inferior parietal lobe (IPL), *p* range = 0.013–0.043 and 3 channels over left IPL, *p* range = 0.019–0.030. There were moderate negative relationships between IPL activity and balance, r range = −0.441–0.419, *p* range = 0.031–0.046. **Conclusions**: We observed significant task-dependent neural activity in superior and inferior parietal lobes. Additionally, greater neural recruitment of the inferior parietal lobes was associated with less sway during balance performance, which provides evidence of the neural underpinnings of balance in ABI.

## 1. Introduction

Worldwide, an estimated 69 million people sustain traumatic brain injuries (TBI) [1] and approximately 12 million people experience stroke [2]. Collectively, TBI and stroke, along with brain tumors, anoxic injuries, and other insults to brain tissue are considered acquired brain injuries (ABI). ABI can cause psychological [3], cognitive [4], and motor impairments, including balance deficits [5]. Importantly, balance deficits may increase the risk of falling and can impact the quality of life of people with ABI [6].

To address balance deficits in adults with ABI, it can be helpful to consider the neural substrates that support balance in healthy individuals. Functional magnetic resonance imaging (fMRI) studies have highlighted the essential role of the cerebellum in balance [7,8,9], but have also indicated that cortical structures, like the primary motor cortex, supplementary motor area, superior inferior parietal lobe (SPL) and inferior parietal lobe (IPL) support balance performance [8]. However, such findings are indirect since fMRI cannot measure task-dependent neural activity during standing tasks, like balance, due to movement limitations [10]. Instead, such findings are usually based on findings during imagined standing or by linking data from brain structures to balance performance collected at a different time point. Other neuroimaging techniques, like functional near-infrared spectroscopy (fNIRS), can measure task-dependent neural activity from superficial cortical structures [11,12], like the parietal lobes, during balance tasks. Thus, the cortical underpinnings of balance performance can be directly evaluated via fNIRS in both healthy and clinical populations.

FNIRS indirectly measures brain activity by sending near-infrared (NIR) light and detecting refracted light from the scalp to quantify changes in oxygenated (HbO) and deoxygenated (HbR) hemoglobin [12,13]. Briefly, following neural activity, HbO increases and HbR decreases compared to baseline values [12,14]. In healthy individuals, Mihara et al. [15] and Takakura et al. [16] have reported increased HbO, with no significant [15] or reported changes in HbR [16], in superficial cortical regions during balance tasks. Mihara et al. [15] found that anterior–posterior (forward–backward) postural perturbations led to increased HbO in the middle frontal gyrus, superior frontal gyrus, precentral gyrus, and SPL during warned (i.e., auditory warning to indicate the start of perturbation) and unwarned tasks. Takakura et al. [16] found increased HbO in the premotor cortex, supplementary motor area, SPL, frontal gyrus, parietal operculum, and superior temporal gyrus. However, in both studies [15,16] it was unclear if there was a relationship between increased HbO and balance performance.

To our knowledge, only one fNIRS study has evaluated task-dependent activity during balance in adults with ABI. Here, researchers first evaluated task-dependent changes in neural activity and then measured the relationship between task-dependent neural activity and balance performance in adults with stroke before any intervention [17]. They found increased HbO, with no significant changes in HbR, in the middle frontal gyrus, superior frontal gyrus, SPL, and precuneus during postural perturbations. They also found positive relationships between HbO in the supplementary motor area and prefrontal cortex activity and balance performance, as measured with the Berg Balance Scale [17]. This indicates that increased neural activity from the supplementary motor area and the prefrontal cortex may be needed to support better balance performance. However, this requires further inquiry as neural activity was not measured during Berg Balance Scale tasks. Rather, neural activity was generated from a separate balance task (forward–backward perturbations) and that neural activity was associated with Berg Balance Scale scores.

As such, there are two primary objectives of this study. The first is to examine frontoparietal neural activity during balance tasks in adults with chronic ABI. The second objective is to examine the relationship between the task-dependent neural activity and balance performance metrics, from those exact balance tasks. We hypothesize that there will be increased HbO and no change in HbR, in superficial cortical regions, and we expect that increased HbO will be associated with better balance performance.

## 2. Materials and Methods

### 2.1. Study Design

This was a secondary data analysis of data derived from a single-blind physical activity randomized control trial (RCT) that measured balance in chronic ABI [18]. These analyses examined baseline or pre-intervention task-dependent neural activity during static balance tasks. Baseline data were acquired 2–3 weeks before the first intervention session. All study procedures were approved by the Institutional Review Board at Colorado State University and participants provided written informed consent.

### 2.2. Participants

Individuals with ABI were recruited using email list serves, newspaper and radio advertisements, physical flyers, quarterly newsletters, and during brain injury or stroke support group meetings. Participants were included in the RCT if they were adults (i.e., 18 years or older), had chronic ABI (≥6 months post-injury), and had self-reported medium-to-severe balance issues as assessed with the Neurobehavioral Symptom Inventory [19]. Participants were excluded if their most recent brain injury occurred less than 6 months before data collection or if they self-reported mild balance or no balance issues. Eligibility of prospective participants was verified via a phone screening which was conducted by trained research personnel; those who met inclusion criteria were scheduled for an initial assessment and enrolled in the study.

### 2.3. Outcome Measures

Outcome measures were completed immediately after consent was obtained. A complete list of outcome measures for the pilot RCT are included in the protocol paper Stephens et al. [18]. For this study, outcome measures were task-dependent neural activity via fNIRS and balance performance via a force plate. These data were acquired simultaneously using a randomized block design (described below).

#### 2.3.1. Task-Dependent Neural Activity

**Figure 1:**
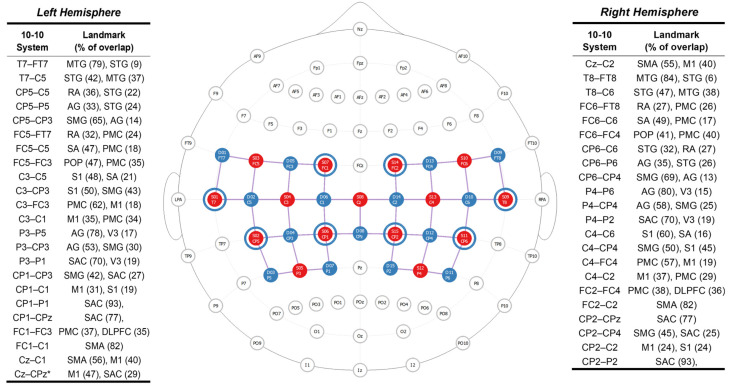
fNIRS head probe. Functional near-infrared spectroscopy head probe and anatomical landmarks from the left and right hemispheres. The head probe has 15 light-emitting optodes (sources—red dots), 15 light detectors (detectors—blue dots), and eight short-separation channels (blue rings around light-emitting diodes). A total of 45 source-detector pairs (channels—purple lines) located over the frontoparietal region were used to collect task-dependent neural activity. The fNIRS head probe is based on the 10-10 EEG coordinate system. with the brain regions associated with each source-detector channel (anatomical landmark) and specificity (% overlap) mentioned based on the Brodmann Atlas from fNIRS Optodes’ Location Decider (Zimeo Morais et al., 2018 [20]). Abbreviations: AG: Angular Gyrus, DLPFC: Dorsolateral Prefrontal Cortex, MTG: Middle Temporal Gyrus, M1: Primary Motor Cortex, PMC Premotor Cortex, POP: Pars Opercularis, RA: Retrosubicular Area, SA: Subcentral Area, SAC: Somatosensory Association Cortex, SMA: Supplementary Motor Area, SMG: Supramarginal Gyrus, STG: Superior Temporal Gyrus, S1: Primary Somatosensory Cortex, V3: Visual Area Three. * Source-detector channel located at midline, between the left and right hemispheres.

These data were acquired at a 4.6 Hz sampling rate with a portable fNIRS device, the NIRSport2 (NIRx Medical Technologies, LCC, Berlin, Germany) and its proprietary software, Aurora v2021.9. Portable fNIRS devices do not provide full head coverage, so regions of interest (ROIs) associated with balance performance were selected a priori. The following ROIs were selected: bilateral supplementary motor area, bilateral primary motor cortex, bilateral superior parietal lobe (supplementary association cortex), and bilateral inferior parietal lobe (angular gyrus and supramarginal gyrus). Then, the placement of 15 light-emitting diodes (LEDs) sources, which emitted light at 760 nm and 850 nm wavelengths, and 15 detectors was determined using the fNIRS Optodes Location Decider (fOLD) toolbox [20]. Additionally, eight short-separation channels (SSC) were used to measure scalp perfusion [21,22]. Sources and detectors were positioned 2.5–3.5 cm apart, while each SSC was placed 0.8 cm apart from the source; see Figure 1.

#### 2.3.2. Balance Performance Data

Balance performance data were collected at a 25 Hz sampling rate with a previously validated force plate [23,24,25], BTrackS (Balance Tracking Systems, Inc., San Diego, CA, USA) and its proprietary software, BTrackS Explore Balance v2.0.4. Participants completed balance tasks while barefoot with arms crossed and feet hips-distance apart on either a firm or soft surface. The firm surface was the force plate, and the soft surface was a foam pad placed on top of the force plate. A total of four static standing balance tasks were included: Eyes Open Firm Surface (EO_Firm_), Eyes Closed Firm Surface (EC_Firm_), Eyes Open Soft Surface (EO_Soft_), and Eyes Closed Soft Surface (EC_Soft_). Balance performance data were collected and quantified as the displacement of the center of pressure (CoP) in anterior–posterior (AP) or medial–lateral (ML) planes. Larger CoP displacement values indicate greater postural sway or instability while standing, so higher values represent poorer balance performance.

### 2.4. Data Acquisition Procedures

**Figure 2:**
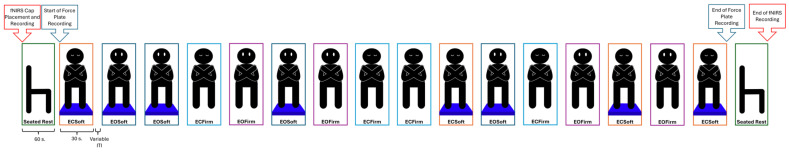
Randomized Block Design. Visual depiction of the a priori randomized order of balance tasks performed by each participant while wearing an fNIRS cap and balancing over the BTrackS force plate. Abbreviations: EC_Soft_: Eyes Closed Soft Surface, EO_Soft_: Eyes Open Soft Surface, EC_Firm_: Eyes Closed Firm Surface, EO_Firm_: Eyes Open Firm Surface, ITI: Inter-trial interval.

After participants signed a consent form, research personnel measured their head circumference and fitted them with an appropriately sized fNIRS cap. Three adult-sized fNIRS caps (54 cm, 56 cm, and 58 cm) were used in this study. After donning the fNIRS cap, a black shower cap was placed over the fNIRS cap to reduce fluorescent light interference and improve signal quality. Next, signal optimization steps were completed in Aurora until good or excellent signal quality was reached at all channels (signal level > 0.5 mV; dark noise > 3.0 µV and <20.0 µV, and coefficient of variation < 7.5%). Following signal optimization, a randomized block design was used to acquire all data, and distinct event markers for balance tasks were integrated into the fNIRS data file via PsychoPy software (v.2012.1.3) [26,27,28] and a lab streaming layer. The block design began and ended with one minute of seated, quiet rest. Between these rest periods, four balance tasks were repeated four times each (total of 16 trials) in a randomized order to prevent neural habituation. The order of tasks was randomized by assigning a number to each task and then using a random order generator to determine a random order. Importantly, this random trial order was consistent across participants to avoid introducing a task-order effect. Each balance trial was 30 s in duration and included variable inter-trial intervals (ITIs); see Figure 2. ITIs were at least 10 s long but often longer for participants who needed rest or extra time for instructions between trials. Although a variable ITI may have introduced some measurement error, it was essential for participant safety.

### 2.5. Task-Dependent Neural Activity Data Processing

fNIRS data were processed using Satori (v2.0, NIRx Medical Technologies, LCC, Berlin, Germany) by uploading each ‘snirf’ file. As files were uploaded, raw signal was automatically converted to optical density and spatial registration of data to the head probe layout was automatically performed by Satori (v2.0, NIRx Medical Technologies, LCC, Berlin, Germany). Then, the event markers generated by PsychoPy were renamed by research personnel using balance task identifiers (e.g., Event 1 = “EC_Soft_”). Next, channel rejection was completed using a scalp coupling index threshold of 0.75 [29]. After this, optical data were converted to hemodynamic concentrations (HbO, HbR, and total hemoglobin) using the Modified Beer–Lambert Law [30]. Next, temporal processing of the signal consisted of motion correction using a spike-removal monotonic interpolation (10 iterations, 3.5 threshold, 5 s lag, and 0.5 influence) and a temporal derivative distribution repair (TDDR) [31]. Then, the data were filtered using a Butterworth band-pass filter with the low-pass set at 0.2 Hz and the high-pass set at 0.01 Hz. The mentioned values of the low-pass and high-pass filters were used after visual examination of the spectrum frequency components of the signal of three randomly selected data files. A generalized linear model (GLM) based short-channel regression using all the short-channels (n = 8) was used to detect and eliminate physiological noise not related to neural activity. All data were baseline-zero adjusted. Finally, beta coefficients were generated using the GLM function for HbO and HbR, per channel and task for each participant.

### 2.6. Balance Performance Data Reduction & Analysis

All data from Individual trials where participants required physical support to avoid falling were excluded from calculations. Then, force plate data were reduced and analyzed using a custom-made MATLAB script that used previously established methods (MATLAB 2023a, The MathWorks, Inc., Natick, MA, USA). In this script, the first five seconds of each balance task were excluded to allow for habituation to the task, as implemented by Richmond et al. [25]; thus, balance metrics were calculated during 25 s. Then, the script calculated three balance performance metrics for each balance trial and participant: center of pressure length (COP_Length_), anterior–posterior range (AP_Range_), and mediolateral range (ML_Range_). Center of pressure length was calculated based on Prieto et al. [32], ${COP}_{Length}=\;\sum_{n=1}^{N-1}{\left[{\left(AP\left[n+1\right]-AP\left[n\right]\right)}^{2}+{\left(ML\left[n+1\right]-ML\left[n\right]\right)}^{2}\right]}^{1/2},\;$ later used by Goble et al. [33]. Anterior–posterior range and medial–lateral range were calculated by subtracting the smallest value from the largest value pertaining to the anterior–posterior and medial–lateral center of pressure data, respectively [32]. After obtaining individual trial balance performance metrics, an averaged COP_Length_, AP_Range_, and ML_Range_ were calculated from the four trials of the four balance tasks (EO_Firm_, EC_Firm_, EO_Soft_, EC_Soft_) for each participant using the *AVERAGE* function from Excel (Microsoft Corporation, Redmond, WA, USA).

### 2.7. Statistical Analysis

To exclude fNIRS channels from participants with superficial lesions over fNIRS’ ROIs, T1-weighted brain scans from a 3T MRI System (MAGNETOM Skyra, Siemens Healthineers, Erlangen, Germany) were visually inspected with Mango Software (v4.1, Research Imaging Institute, UTHSCSA, San Antonio, TX, USA) to partially manage the influence of such lesions on our findings [18,34]. All data were examined for outliers by converting each value to Z-scores and eliminating those values higher than 3 or lower than −3. Additionally, Using JASP v.0.19.0.0 [35], the Shapiro–Wilk test for normality was used to examine all data for potential normality assumption violations. For HbO activity per balance task, a channel-wise one-sample t-test (HbO > 0) was used for normally distributed data. The Wilcoxon signed-rank test was used for data that was not normally distributed. For HbR activity per balance task, a channel-wise one-sample t-test (HbR < 0) was used for normally distributed data. The Wilcoxon signed-rank test was used for data that was not normally distributed. Effect sizes, Cohen’s d for one-sample t-test, and Matched ranked biserial correlation for the Wilcoxon signed-rank test were calculated and reported. Due to multiple statistical tests and to avoid Type I errors, a false discovery rate (FDR) correction set to 0.05 was used [36].

Lastly, Pearson’s correlation was used to examine relationships between statistically significant channels and balance performance metrics (COP_Length_, AP_Range_, and ML_Range_).

## 3. Results

Data were acquired from 27 participants (Age (M ± SD): 51.30 ± 18.67 years). Demographics and brain injury characteristics are provided in Table 1.

### 3.1. Task-Dependent Neural Activity Findings

**Figure 3:**
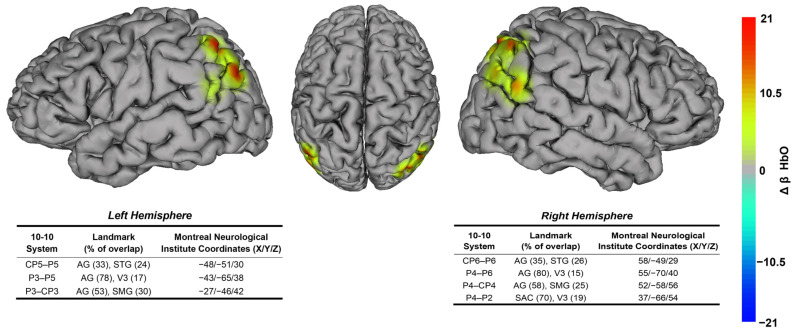
Task-dependent neural activity changes during Eyes Open Soft Surface (EO_Soft_) balance. The heat map overlaid on the brain shows significantly increased neural activity (HbO), which is visually represented by warm colors (yellow and red). The tables below correspond to the statistically significant source-detector channels for each brain hemisphere after false discovery rate (FDR) correction. The brain regions associated with each source-detector channel (anatomical landmark), specificity (% overlap), and Montreal Neurological Institute Coordinates (MNI) mentioned are based on the Brodmann Atlas from fNIRS Optodes’ Location Decider [20]. Abbreviations: AG: Angular Gyrus, SAC: Somatosensory Association Cortex, SMG: Supramarginal Gyrus, STG: Superior Temporal Gyrus, V3: Visual Area Three.

One-sample t-tests revealed that HbO was significantly increased during balance tasks with eyes open (EO_Firm_ and EO_Soft_). During the Eyes Open Soft Surface task, HbO was significantly increased in the P4-P2 channel located over the right somatosensory association cortex (part of SPL); t (22) = 2.75; *p* = 0.039; *d* = 0.574; ꞵ_HbO_: 20.46 ± 35.65, and on several channels located over the left (CP5–P5, P3–P5, and P3–CP3) and right (CP6–P6, P4–P6, and P4–CP4) angular gyrus (part of IPL; *p* range = 0.019–0.038) with effect sizes ranging from 0.551 to 0.765; see Figure 3. During the Eyes Open Firm Surface task, HbO was significantly increased in the CP6–P6 channel located over the right angular gyrus (part of IPL); t (23) = 3.50; *p* = 0.043; *d* = 0.716; ꞵ_HbO_: 15.91 ± 22.24. HbO was not significantly increased in any other channels during any of the eyes open tasks after FDR correction (*p* range = 0.124–1). Also, HbO was not significantly increased during any of the eyes closed tasks after FDR correction (*p* range = 0.09–1). There were no significant changes in HbR in any channel during any of the four balance tasks (*p* range = 0.167–1).

### 3.2. Relationship Between Task-Dependent Neural Activity and Balance

**Figure 4:**
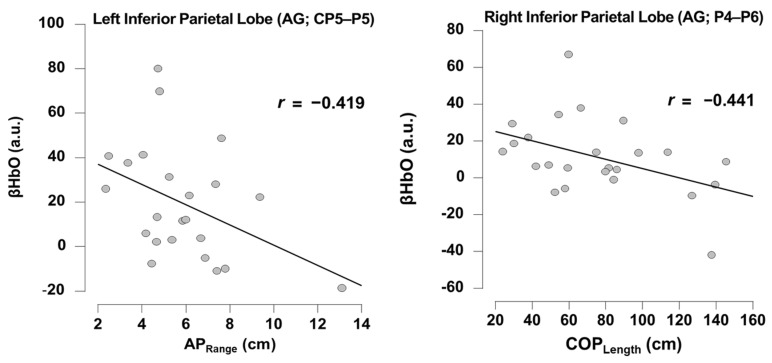
Pearson’s correlation between balance performance metrics and neural activity (HbO) during Eyes Open Soft Surface tasks. Notice that larger anterior–posterior range (AP_Range_) and center of pressure length (COP_Length_) indicate greater postural sway or instability during the balance task and thus worse balance performance. Abbreviations: AG: Angular Gyrus.

Pearson’s correlations revealed there were significant negative relationships during the Eyes Open Soft Surface task. Specifically, there was a negative relationship between neural activity in channel CP5-P5 (left anterior gyrus, part of IPL) and AP_Range_ (r = −0.419; *p* = 0.046). Also, there was a negative relationship between neural activity in channel P4-P6 (right anterior gyrus, part of IPL) and COP_Length_ (r = −0.41; *p* = 0.031); see Figure 4. None of the other channels showed any significant relationship with any of the balance performance metrics (*p* range = 0.115–0.994). Pearson’s correlations revealed there were no significant relationships during Eyes Open Firm Surface task between neural activity in channel CP6-P6 (right anterior gyrus, part of IPL) and balance performance metrics (COP_Length_, AP_Range_, ML_Range_) (*p* range: 0.813–0.839).

## 4. Discussion

This study was designed to examine frontoparietal neural activity during balance tasks in adults with chronic ABI and to examine the relationship between the task-dependent neural activity and balance performance metrics. For our first objective, we hypothesized that there would be increased HbO and no change in HbR during the four different balance tasks. This hypothesis was supported, as we found that HbO increased over the right SPL, and right and left IPL during eyes open tasks. Additionally, and as observed in other studies [15,17,37], there was no significant change in HbR during any of the balance tasks.

These findings are logical given our understanding of neural underpinnings of balance in healthy individuals. Goble et al. [38] observed bilateral activation of IPL during tendon vibration of the ankle joint, while no IPL activation was reported during vibration of the bones. Since tendon vibration stimulates sensory receptors (e.g., muscle spindles) [39], IPL activity during vibration likely reflects sensory information from the muscle. In this study, our findings showed increased HbO over IPL, which might reflect sensory information from lower limb muscles during balance tasks with the eyes open. As documented in Pellijeff et al. [40] and Naito et al. [41], the SPL is involved in integrating sensory information from different body parts to generate and update the body representation of its interaction with the space and external objects. Thus, in our study, the findings on increased HbO on SPL may reflect the integration of sensory information from the body limbs as it relates to space and its interaction with the foam pad when eyes were open. Importantly, other authors, using fNIRS, have reported increased HbO over SPL during different balance tasks in healthy populations [15,16] and in people with stroke [17].

Together, the IPL and SPL, as part of the posterior parietal cortex (PPC), may help integrate sensory information from the body and its interaction with external objects, as is the case with balance tasks over a force plate. It is unclear why there were no significant HbO changes during balance tasks with the eyes closed in the posterior parietal cortex. However, Takakura et al. [16] reported similar findings, as increased HbO in PPC was observed during balance tasks with the eyes open, but no differences in PPC were observed during tasks with the eyes closed. Interestingly, during illusory wrist flexion from vibration, the PPC is more active when visual information is present (e.g., video of the vibrated hand) [42]. This could explain why, in this study, HbO from the PPC was significantly higher during eyes-open tasks but did not increase significantly during balance tasks with eyes closed.

### 4.1. Balance Performance Relates to Posterior Parietal Cortex Neural Substrates

We also sought to explore the relationship between the task-dependent frontoparietal neural activity and balance performance. We observed multiple significant relationships between ROIs with increased HbO activity and balance performance. Specifically, increased HbO in the left IPL was associated with less sway, or less postural instability, in the anterior–posterior plane. Additionally, increased HbO activity in the right IPL angular gyrus was associated with less sway in the center of pressure.

Again, findings from previous studies support the interpretation of these results. Goble et al. [38] examined the relationship between brain activity and anterior–posterior equilibrium scores during a balance task with the eyes closed and a fixed platform. Goble et al. [38] found a positive relationship between the right IPL and the anterior–posterior equilibrium score, indicating that more IPL neural activity supported better balance performance, although this work was completed with fMRI which did not support measurement of task-dependent neural activity during balance tasks.

To our knowledge, aside from our study, there is only one other study examining the relationship between task-dependent neural activity and balance in people with stroke (a type of ABI) at baseline [17]. Mihara et al. [17] reported that higher HbO activity in the supplementary motor area and the prefrontal cortex were associated with better balance performance. Interestingly, although Mihara et al. [17] reported increased HbO in SPL during postural perturbations, SPL activity was not correlated to balance performance as measured with the Berg Balance Scale. Given methodological differences between studies, it is unknown if SPL activity would have been associated with anterior–posterior sway, as we observed. Also, unlike our study, Mihara et al. [17] did not use short-separation channels to measure scalp perfusion; thus, the lack of similar findings might be due to fNIRS acquisition differences.

### 4.2. Limitations and Future Directions

The data reported in this study are from 27 participants (18 female and 9 male) whose ages ranged from 19 to 82 years old. Most of them (n = 21) self-reported moderate loss of balance, and 41% of all the participants indicated ‘not currently being involved in any rehabilitation.’ From an injury characteristic (e.g., time-since-injury) and age perspective, our findings are based on a heterogeneous sample, and we were not adequately powered to evaluate how these characteristics influenced our findings, so our results should be interpreted with caution. Additionally, all our participants were homogeneous from a racial and ethnic perspective, which limits generalizability. Moreover, due to the primary objectives of the pilot RCT, we did not include healthy controls. In future studies, potential confounding variables (e.g., time-since-injury and age) should be evaluated with a larger sample size. Also, it would be interesting to compare task-dependent neural activity between healthy controls and people with ABI to enhance our understanding of neurophysiology following brain injury. As another notable limitation, during data collection, some, but not all, participants experienced fatigue. It is still unclear to what extent fatigue could have influenced the participants’ hemodynamic response or mental state. This is almost inevitable with a clinical population, but fNIRS acquisition methods could be shortened to reduce fatigue. Lastly, subcortical areas of the brain may have been actively involved in balance; however, it was not possible to examine such areas due to inherent limitations (i.e., limited depth of measurement) of fNIRS [43]. Future studies could take advantage of multimodal approaches, such as using fMRI and fNIRS approaches in tandem.

## 5. Conclusions

Here, we found increased neural activity of the IPL and SPL during eyes-open balance tasks in adults with ABI. The increased activity of IPL and SPL underscore their importance in sensory integration to facilitate balance control when the eyes are open. Furthermore, the observed relationships between higher HbO activity from IPL and less sway (i.e., postural instability) suggest that increased neural activity from IPL may support better balance performance. These findings could have implications for rehabilitation approaches for individuals with ABI. For example, sessions of non-invasive stimulation over IPL (i.e., transcranial magnetic stimulation), in addition to sensorimotor integration exercises, may help to improve balance in people with chronic ABI. However, more research is needed to improve our understanding of task-dependent brain activity after ABI and how neural activity supports or inhibits functional tasks.

## Figures and Tables

**Table 1:** Participants’ Demographics and Brain Injury Characteristics.

	All (N = 27)	Female (n = 18)	Male (n = 9)
	Mean ± SD (Min–Max) or N (%)	Mean ± SD or n (%)	Mean ± SD or n (%)
Age (years)	51.30 ± 18.67 (19.00–82.00)	50.22 ± 16.14	53.44 ± 23.91
Time Since Most Recent Injury (years)	13.30 ± 17.38 (0.50–62.00)	15.51 ± 19.48	8.89 ± 11.93
Current Time in Rehabilitation (years)	0.88 ± 1.33 (0.00–5.00) *	0.78 ± 1.18 *	1.06 ± 1.63
Ethnicity (not Hispanic)	26 (96.30)	17 (94.44)	9 (100.00)
Race (white)	27 (100.00)	18 (100.00)	9 (100.00)
Brain Injury Type			
Non-TBI	11 (40.74)	6 (33.33)	5 (55.56)
TBI	14 (51.85)	11 (61.11)	3 (33.33)
Both	2 (7.41)	1 (5.56)	1 (11.11)
Educational Level			
High School	3 (11.11)	0 (0.00)	3 (33.33)
Some College	8 (29.63)	5 (27.78)	3 (33.33)
College Graduate	8 (29.63)	7 (38.89)	1 (11.11)
Some Post-graduate	1 (3.70)	1 (5.56)	0 (0.00)
Post-graduate Degree	7 (25.93)	5 (27.78)	2 (22.22)
Loss of Balance			
Moderate	21 (77.78)	16 (88.89)	5 (55.56)
Moderate to Severe	3 (11.11)	2 (11.11)	1 (11.11)
Severe	3 (11.11)	0 (0.00)	3 (33.33)

* Descriptive statistics based on 25 participants (Female n = 16).

## Data Availability

The data presented in this study are available on request from the corresponding authors to protect participant confidentiality (specify the reason for the restriction).

## Abbreviations

The following abbreviations are used in this manuscript:

ABI	Acquired Brain Injury
AP	Anterior–Posterior
AP_Range_	Anterior–Posterior Range
CoP	Center of Pressure
COP_Length_	Center of Pressure Length
EC_Firm_	Eyes Closed Firm Surface
EC_Soft_	Eyes Closed Soft Surface
EO_Firm_	Eyes Open Firm Surface
EO_Soft_	Eyes Open Soft Surface
FDR	False Discovery Rate
fMRI	Functional Magnetic Resonance Imaging
fNIRS	Functional Near-infrared Spectroscopy
fOLD	FNIRS Optodes Location Decider
GLM	Generalized Linear Model
HbO	Oxygenated Hemoglobin
HbR	Deoxygenated Hemoglobin
IPL	Inferior Parietal Lobe
ITI	Inter-trial Interval
LED	Light-emitting Diode
ML	Medial–Lateral
ML_Range_	Medio-Lateral Range
RCT	Randomized Control Trial
ROI	Region of Interest
SPL	Superior Parietal Lobe
SSC	Short-separation Channel
TBI	Traumatic Brain Injury
TDDR	Temporal Derivative Distribution Repair
